# Is non-invasive neuromodulation a viable technique to improve neuroplasticity in individuals with acquired brain injury? A review

**DOI:** 10.3389/fnhum.2024.1341707

**Published:** 2024-09-04

**Authors:** Michelle Eliason, Prajakta Premchand Kalbande, Ghazala T. Saleem

**Affiliations:** Rehabilitation Science Department, University at Buffalo, Buffalo, NY, United States

**Keywords:** transcranial electrical stimulation, transcranial magnetic stimulation, brain injuries, cerebral stroke, neurological rehabilitation

## Abstract

**Objective:**

This study aimed to explore and evaluate the efficacy of non-invasive brain stimulation (NIBS) as a standalone or coupled intervention and understand its mechanisms to produce positive alterations in neuroplasticity and behavioral outcomes after acquired brain injury (ABI).

**Data sources:**

Cochrane Library, Web of Science, PubMed, and Google Scholar databases were searched from January 2013 to January 2024.

**Study selection:**

Using the PICO framework, transcranial magnetic stimulation (TMS) and transcranial direct current stimulation (tDCS) randomized controlled trials (RCTs), retrospective, pilot, open-label, and observational large group and single-participant case studies were included. Two authors reviewed articles according to pre-established inclusion criteria.

**Data extraction:**

Data related to participant and intervention characteristics, mechanisms of change, methods, and outcomes were extracted by two authors. The two authors performed quality assessments using SORT.

**Results:**

Twenty-two studies involving 657 participants diagnosed with ABIs were included. Two studies reported that NIBS was ineffective in producing positive alterations or behavioral outcomes. Twenty studies reported at least one, or a combination of, positively altered neuroplasticity and improved neuropsychological, neuropsychiatric, motor, or somatic symptoms. Twenty-eight current articles between 2020 and 2024 have been studied to elucidate potential mechanisms of change related to NIBS and other mediating or confounding variables.

**Discussion:**

tDCS and TMS may be efficacious as standalone interventions or coupled with neurorehabilitation therapies to positively alter maladaptive brain physiology and improve behavioral symptomology resulting from ABI. Based on postintervention and follow-up results, evidence suggests NIBS may offer a direct or mediatory contribution to improving behavioral outcomes post-ABI.

**Conclusion:**

More research is needed to better understand the extent of rTMS and tDCS application in affecting changes in symptoms after ABI.

## Introduction

Acquired brain injury (ABI) includes traumatic brain injury (TBI) and non-traumatic brain injury (nTBI). TBIs are caused by an external event (e.g., motor vehicle accident), while an nTBI results from an internal process leading to cerebral damage (e.g., stroke) (Assecondi et al., 2020; Goldman et al., 2022). In the United States, approximately 1.5 million people survive an ABI each year (Georges, 2023), and 30% of survivors will continue to experience symptoms that may disrupt everyday activities (Allonsius et al., 2023). The sustained trauma disrupts neural connections, leading to physical impairments (e.g., movement deficits and pain), somatic symptoms (e.g., fatigue), neuropsychological dysfunction (e.g., impaired executive function (EF), memory loss, and arousal), and neuropsychiatric dysfunction (e.g., social and mental health). Physical therapy (PT), occupational therapy (OT), and cognitive therapy (CT) are rehabilitation techniques that have improved outcomes for individuals with ABI (Scherer, 2007; Cernich et al., 2010; Ustinova et al., 2015; Mikolić et al., 2019). When combined with OT and PT, CT addresses aspects of behavioral function to produce a synergistic effect on neuroplasticity (Embrechts et al., 2023). While these broadly recognized therapies hold a central place in neurorehabilitation for their value, the effectiveness of many neurological interventions varies significantly due to temporal constraints, personal factors, financial feasibility, and other barriers (Clayton et al., 2016; Dang et al., 2017; Hofer and Schwab, 2019; Diaz et al., 2023). For example, constraint-induced movement therapy (an effective intervention for ABI; CIMT) requires intensive dosage (e.g., 6 h) to facilitate effective extremity function (Cimolin et al., 2012; Reiss et al., 2012; Pedlow et al., 2014). Due to some of these barriers, many strongly supported interventions have not been widely incorporated into clinical practice (Fleet et al., 2014). Thus, a critical need remains for a neurorehabilitation treatment approach that integrates therapeutic approaches to extend neurorehabilitation treatment’s efficacy, dosage, and number of responders.

There is a growing interest in the efficacy of non-invasive brain stimulation (NIBS) as a complement or supplement intervention to current approaches in ABI recovery to address bottom-up implications, including inflammation, edema, disruption of the blood–brain barrier (BBB), white matter destruction, excitotoxicity, and damage to vasculature as well as top-down implications including cognitive deficits, mood dysregulation, and occupational performance deficits (Villamar et al., 2012; Pope and Miall, 2014; Wessel et al., 2015; Clayton et al., 2016; Dang et al., 2017; Hofer and Schwab, 2019; Cha and Hwang, 2022; Nousia et al., 2022; Diaz et al., 2023). By modulating neural activity in specific brain regions, NIBS may promote synaptic and structural neuroplasticity by decreasing cortical hyperexcitability, enhancing long-term synaptic plasticity to avoid maladaptive consequences, facilitating the formation of new neural connections, and facilitating promoting cortical reorganization and consolidation of learning when coupled with physical, cognitive, or behavioral therapies (Bolognini et al., 2009; Schlaug et al., 2011; Villamar et al., 2012; Fregni et al., 2015).

The NIBS methods such as repetitive transcranial magnetic stimulation (rTMS) and transcranial direct current stimulation (tDCS) involve using magnetic or electrical fields to modulate neural activity in specific brain regions. rTMS delivers a series of magnetic pulses to the same brain region over time and can be divided between high frequency (HF-rTMS) using ≥ 5 Hz and low frequency (LF-rTMS) using ≤1Hz. tDCS applies low-level direct electrical current to the scalp to modulate cortical excitability through anodal (a-tDCS), widely associated with an excitatory response, or cathodal (c-tDCS), which is associated with an inhibitory response (Goldman et al., 2022).

Despite NIBS’ use to address ABI-related mechanisms of injury for over a decade, a mechanistic model for neuroplasticity has yet to be established. Many recent studies, commentaries, and literature reviews being published report only on the physical symptom and behavioral impact of NIBS (Giordano et al., 2017; Schwertfeger et al., 2023; Zotey et al., 2023; Galimberti et al., 2024) or rationalize the mechanism of change to singular mechanisms such as the specific montage and polarity of tDCS electrodes (anode or cathode) to provide either an excitatory or inhibitory impact (Asloun et al., 2008; Bikson et al., 2010; Calderone et al., 2024), the ability for tDCS to influence resting membrane potential of the neuron (Chang et al., 2023; Calderone et al., 2024), and the inhibitory or excitatory impact of rTMS based on frequency and duration of exposure (Eldaief et al., 2013; Chang et al., 2023; Evancho et al., 2023; Calderone et al., 2024). Although these principles serve as an essential foundation for the study of NIBS post-ABI, anodal and cathodal stimulation in tDCS (Giordano et al., 2017) and duration and frequency of exposure in rTMS (Huang et al., 2005) are not synonymous with excitatory and inhibitory stimulation concerning their effects on neural function and human behavior (Giordano et al., 2017), nor do these rationales provide a potential model for uniformity among future study designs related to NIBS.

Many variables impact stimulation response, including pathological characteristics and pathogenesis of the primary diagnosis (e.g., type of injury, location of injury, and time since injury), person-specific variables (e.g., individual neuroanatomy, genetic factors, and current medication), and NIBS methodology (e.g., dosing parameters, electrode size, duration of the stimulation, current density, and simultaneous activities being performed with NIBS) (Zettin et al., 2021). These many considerations make it imperative to establish a model of neuroplasticity that communicates the pathophysiological complexities of NIBS’ impact on cortical and subcortical structures and allows for an improved understanding of its mechanism of action within the brain and its effect on behavior. We propose hormesis-based neuroplasticity as a potential mechanistic model to guide and support the application of NIBS post-TBI.

Hormesis is a biphasic dose–response (DR) model that explains the physiological process whereby cells can respond to targeted, low-level environmental challenges (i.e., tDCS and rTMS) in a manner that subsequently increases their capacity for resilience and functional abilities, resulting in an improved ability to respond in ways that prepare them to resist and recover better from future challenges, including brain damage (Mattson and Leak, 2024). The hormetic dose–response curve typically includes both beneficial effects at low doses and detrimental effects at high doses. In the context of ABI, the focus is on the beneficial adaptations that occur within the ‘hormetic zone’ (Mattson and Leak, 2024). These adaptations, often called “stress-modulated, enhanced metabolic states of cells, involve improved energy, material, and information processing” (Leak et al., 2018). In particular, information processing has been suggested as a primary symptom of mild TBI (mTBI) sequelae (Senathi-Raja et al., 2010; Dymowski et al., 2015). This concept has been extensively studied in ischemia research, where mild ischemic episodes can improve biological defenses and reduce damage from subsequent severe ischemic events (Stetler et al., 2014; Mattson and Leak, 2024).

Post-ABI, TMS, and tDCS can intermittently challenge the brain, promoting adaptive plasticity without overwhelming it. This controlled stimulation can mitigate maladaptive processes such as chronic neuroinflammation and excitotoxicity, common in TBI, thereby creating a supportive environment for brain repair and adaptation (Calderone et al., 2024; de Macedo Filho et al., 2024). Hormetic principles can elucidate the mechanistic underpinnings of NIBS through its focus on (1) establishing an optimal stimulatory dose (including frequency, intensity, duration, and pulse characteristics) for each individual, (2) identifying the specific brain sites and networks to be targeted, and (3) establishing an understanding of specific cellular components that mediate the stimulatory response (Giordano et al., 2017).

Considering hormetic principles of neuroplasticity and the critical need to develop an understanding of the mechanistic underpinnings of NIBS, the goals of this review are fivefold: (1) Consider hormesis-based neuroplasticity as a potential mechanism of action enhancing neuroplasticity with NIBS; (2) examine cortical excitability, regional cerebral blood flow (rCBF), and white matter integrity as key factors substantiating the use of NIBS as a viable and effective approach for enhancing positive alterations in neuroplasticity; (3) consider differences between NIBS as a standalone treatment and when combined with neurorehabilitation therapies to impact behavioral outcomes (coupled); (4) explore NIBS’ candidacy as the primary mechanism of change affecting behavioral outcomes post-ABI while identifying barriers mitigating this potential and finally; and (5) offer direction for future research.

## Materials and methods

### Study selection

We used the Cochrane Library, Web of Science, PubMed, and Google Scholar databases to identify studies from January 2013 to January 2024. To cast a wide net, a set of key search terms was employed, including “Neuroplasticity,” “Brain Injury,” “Acquired Brain Injury,” “Brain Injury rehabilitation,” Non-invasive brain stimulation,” “TMS,” and “tDCS.” The search was conducted following the PICO framework. It included ‘P’ (patient/problem), i.e., people with ABI, ‘I’ (intervention) with NIBS, ‘C’ (comparison) standalone intervention versus NIBS coupled with other neurorehabilitation therapies, and ‘O’ (outcome) with positive alterations in neuroplasticity and behavioral outcomes (Schardt et al., 2007). The inclusion criteria were as follows: (1) English language, (2) using NIBS as a standalone or coupled intervention, and (3) inclusion of neuroplasticity and behavioral outcomes. The exclusion criteria were as follows: (1) review articles and meta-analyses, (2) case studies without quantitative data, (3) book chapters, and (4) abstracts.

### Quality assessment

The quality of literature was critically analyzed according to Strength of Recommendation Taxonomy (SORT) guidelines where Level 1 is assigned the letter ‘A’ (consistent and good quality patient-oriented evidence), Level 2 is assigned the letter ‘B’ (inconsistent or limited quality and patient-oriented evidence), and Level 3 is assigned the letter ‘C’ (consensus, usual practice, opinion, disease-oriented evidence, and case series of diagnosis and treatment) (Ebell et al., 2004).

### Data extraction

The following results were independently extracted by two authors: (1) metadata (publication date and authorship); (2) participant characteristics (sample size and diagnosis); (3) methods (study design, whether NIBS was used as a standalone treatment or coupled with an additional neurorehabilitation therapy, any outcome measure evaluating treatment efficacy); (4) characteristics of NIBS (current intensity, duration, current type, electrode placement, and number of sessions); and (5) information related to mechanisms of change of NIBS and other mediating and confounding variables.

## Results

### Study characteristics

Of the initial 41 articles identified, 22 were selected according to inclusion criteria. Utilizing PICO, (‘P’), all studies included individuals diagnosed with an ABI; (‘I’) 11 studies investigated tDCS, whereas 11 studies investigated rTMS; (‘C’) 11 studies on tDCS included 264 participants where 10 used a coupled design and 1 used a standalone approach. 11 studies on rTMS included 393 participants, where 4 articles used a coupled intervention and 7 articles used a standalone intervention. (See Table 1); (‘O’) all studies reviewed outcomes of either neuroplasticity or behavioral measures (Rushby et al., 2021; Ulrichsen et al., 2022). Level 1 included 12 randomized controlled trials (RCTs). Level 2 included two retrospective studies, one pilot study, one open-label, prospective case series investigation, and one large group case study with control. Level 3 included two case reports. A total of four non-interventional studies, two explanatory articles, six pre-clinical animal studies, five literature reviews or meta-analyses, and one abstract were eliminated according to exclusion criteria.

**Table 1:** Study characteristics.

	#	Modality	Structure	Design	SORT	Participants with ABI
Middleton et al. (2014)	1	tDCS	Coupled	Consideration-of-concept pilot study	B	*n* = 5
Kurtin et al. (2021)	2	tDCS	Coupled	Large group case study with control	B	*n* = 33
Rushby et al. (2021)	3	tDCS	Coupled	Single-blind, randomized, within-group, cross-over design	A	*n* = 30
Ulam et al. (2015)	4	tDCS	Standalone	Randomized double-blind trial	A	*n* = 26
McCambridge et al. (2018)	5	tDCS	Coupled	Double-blinded and session order randomized.	A	*n* = 10
Ulrichsen et al. (2022)	6	tDCS	Coupled	A randomized sham‐controlled trial combining tDCS with computerized cognitive training	A	*n* = 74
Sacco et al. (2016)	7	tDCS	Coupled	Randomized Control Trial	A	*n* = 32
Eilam-Stock et al. (2021)	8	tDCS	Coupled	Case Study: open label tDCS protocol testing	C	*n* = 1
Quinn et al. (2022)	9	tDCS	Coupled	Double blind, randomized control Trial	A	*n* = 34
Lima et al. (2023)	10	tDCS	Coupled	Double blind, CONTROLLED study	B	*n* = 1
Lefebvre et al. (2013)	11	tDCS	Coupled	Randomized, crossover, placebo controlled, double-blind trial	B	*n* = 18 tDCS=264
Neville et al. (2019)	12	rTMS	Standalone	Randomized double-blind trial	A	*n* = 30
Hara et al. (2017)	13	rTMS	Coupled	Case report	C	*n* = 2
Hara et al. (2016)	14	rTMS	Coupled	Retrospective study; no control	B	*n* = 25
Rodrigues et al. (2020)	15	rTMS	Standalone	A post-hoc analysis of a randomized clinical trial	A	*n* = 27
Siddiqi et al. (2019)	16	rTMS	Standalone	Randomized, controlled, double-blinded pilot study	A	*n* = 15
Rao et al. (2019)	17	rTMS	Standalone	A randomized sham-controlled pilot study	A	*n* = 30
Leung et al. (2016)	18	rTMS	Standalone	Randomized controlled study	A	*n* = 24
Franke et al. (2022)	19	rTMS	Standalone	Randomized controlled study	A	*n* = 28
Zhou et al. (2021)	20	rTMS	Coupled	Retrospective study; with controls	A	*n* = 166
Moussavi et al. (2019)	21	rTMS	Standalone	Randomized, placebo controlled, double-blind study	A	*n* = 22
Luk et al. (2022)	22	rTMS	Coupled	Double-Blind randomized controlled trial	A	*n* = 24 rTMS=393

### Repetitive transcranial magnetic stimulation (rTMS)

Four studies utilized a coupled approach of rTMS spanning 11–15 days to affect change in neuropsychological symptoms (Hara et al., 2016, 2017), motor function (Hara et al., 2016; Luk et al., 2022), and brain perfusion (Hara et al., 2017). One of these studies, a retrospective study, of those with either left (*n* = 10) or right (*n* = 15) post-stroke unilateral upper limb hemiparesis used LF-rTMS over the primary motor cortex (M1) combined with intensive OT (iOT) for 15 days. The results found right LF-rTMS had significant increases in motor and cognitive skills: Fugyl–Meyer Assessment (FMA) categories A–C scores (*p* < 0.05) and Trail Making Test (TMT) (*p* < 0.05), but left LF-rTMS did not have significant results (Hara et al., 2016). Another study investigated two distinct cases. The first case study (diffuse axonal injury (DAI)) used HF-rTMS over the anterior cingulate cortex (ACC). In contrast, the second case study (middle cerebral artery infarction) used LF-rTMS over the dorsolateral prefrontal cortex (DLPFC) and parietal cortex (Hara et al., 2017). Both case studies observed improvements across measures (Schardt et al., 2007) (see Supplementary Table S1), as well as an increase in regional cerebral blood flow (rCBF) over stimulated areas (Hara et al., 2017). The remaining seven RCTs (*N* = 175) used a standalone approach over 5 to 20 consecutive days. Only one study investigating chronic DAI using HF-rTMS over left DLPFC (*n* = 30) failed to find significant improvements across fine motor and neuropsychological testing (Neville et al., 2019). In three rTMS studies over the DLPFC, two used HF-rTMS and one used LF-rTMS to improve neuropsychological and neuropsychiatric symptoms (Rao et al., 2019; Rodrigues et al., 2020; Franke et al., 2022). Although they did not have significant changes in anxiety scores, Rodrigues et al. (2020) found there was a significant change in depression scores (*p* = 0.002) and EF index (*p* = 0.001)(Rodrigues et al., 2020). In an RCT with a crossover design (*n* = 28), there were no significant neuropsychological improvements, but a significant improvement in all neuropsychiatric-related symptoms [e.g., neurobehavioral symptom inventory (*p* = 0.030)] was noted (Franke et al., 2022). The study utilizing LF-rTMS provided mixed results favoring the sham treatment for overall improvement of neuropsychiatric assessments and neuropsychological assessments for delayed recall, cognitive flexibility, and processing speed, whereas the treatment group was favored for neuropsychological tests assessing immediate recall and inhibited automated responses. In this same study of 30 participants, 26 underwent structural magnetic resonance imaging (MRI) with diffusion tensor imaging (DTI) sequences preintervention to assess changes in white matter (WM) connectivity; 19 of those 26 underwent postintervention imaging. The pre–post-comparison of fractional anisotropy (FA) revealed little difference in WM between the groups (Rao et al., 2019). An RCT investigating a major depressive episode secondary to TBI (*n* = 15) used bilateral stimulation for 20 sessions where the initial stimulation targeting the DLPFC node was conducted with left-sided HF-rTMS (4,000 pulses at 10 Hz frequency with 5-s trains and 20-s inter-train interval) followed by right-sided LF-rTMS (a single train of 1,000 pulses with 1 Hz frequency); the antidepressant response was negatively correlated with baseline functional connectivity (FC) between the right-sided stimulation site and the subgenual ACC (sgACC) (*p* = 0.04) within the active group (Siddiqi et al., 2019). Finally, another RCT (*n* = 24) investigated the ability of HF-rTMS over the left motor cortex to decrease neuropsychiatric symptoms, such as headaches, and improve attention (Leung et al., 2016). A higher percentage of the subjects in the active stimulation group (58.3%) significantly showed at least a 50% headache intensity reduction (*p* = 0.035) at post-treatment 1-week assessment compared with the sham group (16.6%)(Leung et al., 2016).

### Transcranial direct current stimulation (tDCS)

Ten tDCS studies used a coupled approach. One double-blinded TBI study (*n* = 26) used a standalone method to investigate whether a-tDCS (1 mA x 20 min) over the left DLPFC would improve attention and memory as measured by alterations in delta and theta waves observed on an electroencephalogram (EEG) over 10 consecutive days (Ulam et al., 2015). Statistically significant results found alterations in theta, delta, and alpha waves with treatment. The delta wave was significantly correlated with improved performance on neuropsychological batteries, such as elevator count with reversal (*p* = 0.006) in the a-tDCS group, compared to no significant changes in the sham group (Ulam et al., 2015). One RCT study of chronic stroke participants (*n* = 10) investigated corticomotor excitability and motor function using a-tDCS (1 mA x 15 min) over the contralesional M1 coupled with a circling task over three sessions; a-tDCS increased corticomotor excitability of both hemispheres trending toward improved paretic intralimb coordination, whereas c-tDCS had no effect (McCambridge et al., 2018). Two RCTs and one case study used a coupled approach with patients diagnosed with a TBI using a-tDCS over the left DLPFC (Sacco et al., 2016; Eilam-Stock et al., 2021; Quinn et al., 2022). Another RCT (*n* = 32) provided a-tDCS (2 mA x 20 min) followed by divided attention (DA) training 2x/day for 5 consecutive days. For both RCTs, active a-tDCS significantly improved reaction time (*t* = 3.41; *p* = 0.004), and fewer omission errors (*t* = 4.49; *p* < 0.0001) were observed in the experimental group on the divided attention (DA) subtest for the examination of attention (TEA), which was maintained after 1 month, compared to no significant improvement in control (Sacco et al., 2016). Another study (*n* = 34) provided a-tDCS (2 mA x 30 min) with simultaneous cognitive training for 10 consecutive weekdays and found both active and control groups demonstrated improvements in EF and post-traumatic symptoms from baseline to 1 month. Anodal tDCS was associated with greater improvements in working memory compared to control (*p* = 0.007) (Quinn et al., 2022). The case study applied a-tDCS (2 mA x 20 min) with simultaneous computerized cognitive training over 20 daily sessions (4 weeks) and found significant post-treatment improvements across neuropsychological and neuropsychiatric measures (Eilam-Stock et al., 2021). Two single-session studies (85 participants) used a-tDCS (1.8 mA-2.0 mA) with simultaneous cognitive therapy. One used an n-Back test (Rushby et al., 2021), and the other used the choice reaction test (Kurtin et al., 2021; Rushby et al., 2021). One study used a tDCS-fMRI (functional MRI) paradigm combining TBI patients and health controls to determine the influence of WM structure on the physiological effects of stimulation using DTI. It concluded that in the absence of task, neither anodal nor cathodal stimulation influenced dorsal anterior cingulate cortex (dACC)/pre-supplementary motor area (preSMA) FA on brain activity. Conversely, during task performance, there was an inverse relationship between WM structure and brain network activity with tDCS; as FA increased, there was more left IFG deactivation (rs = 0.433, *p* = 0.001)(Kurtin et al., 2021). The other study found significant correlations between a-tDCS, decreased arousal and reaction time, diminished performance on a 1-back task, and no effects on the Hospital Anxiety and Depression Scale (HADS), Fatigue and Alertness Scales, nor three Profile of Mood States (POMS) subscales: Vigor, Fatigue, and Depression (all *p* > 0.05)(Rushby et al., 2021). Two studies investigated tDCS efficacy in stroke survivors (n = 59) (Middleton et al., 2014; Ulrichsen et al., 2022). One of these studies used a-tDCS over the ipsilesional motor cortex and c-tDCS over the contralesional cortex (both 1.5 mA x 15 min) with guided motor-based activities over 24 sessions. Results found a clinically meaningful difference in the FMA assessment (mean change = 7.6, effect size = 0.47) (Middleton et al., 2014), and the other of these studies used a-tDCS over DLPFC and c-tDCS over the occipital cortex (both 1 mA x 20 min) with concurrent cognitive training over six sessions. A significant reduction in depression and fatigue symptoms over time was noted, with no significant immediate changes (*p* = 0.021) (Ulrichsen et al., 2022).

## Discussion

### Hormesis-based neuroplasticity and brainstem involvement in NIBS post-ABI

One possible mechanistic model of neuroplasticity that explains the efficacy of NIBS to promote positive physiological changes within cortical and subcortical structures and behavioral impact after ABI is brainstem activation in response to low-grade, targeted stimulation (Mattson and Leak, 2024). Dominant theories of TBI have historically considered the brainstem a primary site of injury because even mild acceleration/deceleration forces can cause a loss of consciousness (LOC), implicating the role of the brainstem in such events (Ward, 1958; Ward, 1964). Even without LOC or notable changes in the cortex, diffuse degeneration of white matter secondary to the shearing of neurons and blood vessels has been observed in other regions, including the brainstem (Strich, 1961; Crompton, 1971; Zimmerman et al., 2023). This degeneration impacts neurotransmitter-producing nuclei like the raphe nuclei (responsible for serotonin production), locus coeruleus (LC) (responsible for norepinephrine production), and pedunculopontine nucleus (PPN), and laterodorsal tegmental nucleus (LTN) (responsible for acetylcholine production) (Parvizi and Damasio, 2003; Valko et al., 2016). These neurotransmitters have a modulatory impact on synaptic behavior within glutamatergic (excitatory) and GABAergic (inhibitory) neurons (Zotey et al., 2023; Mattson and Leak, 2024). They can negatively impact the function of brain systems and networks essential for occupational performance (Giordano et al., 2017).

One implicated system is the reticular activating system (RAS), which receives sensory inputs from various sources and is primarily responsible for arousal, wakefulness, and attention (Ward, 1958). ABI can lead to changes in neurotransmitters such as acetylcholine and serotonin levels, which are crucial for the functioning of the RAS (Sachs, 1957). Increased acetylcholine and serotonin levels in the cerebrospinal fluid post-concussion (Bornstein, 1946; Sachs, 1957) might disrupt normal neurotransmission within the RAS, contributing to the block of sensory conduction. This phenomenon can result in commonly experienced symptoms post-ABI, including attention deficits, low arousal, and fatigue (Zhou et al., 2021), often concomitant with delayed information processing speed and working memory impairments in this population (Sacco et al., 2016). Hormesis-based neuroplasticity offers a potential mechanistic model to explain these adaptations. Specifically, one theory of adaptation suggests that a decrease in RAS function leads to a greater reliance on classical lemniscal pathways, which are less vulnerable to injury. These pathways are more resilient to damage originating at the cortical level, allowing them to continue transmitting sensory impulses to the thalamus and sensory cortex (Foltz and Schmidt, 1956).

Recently, attention has been given to the influence of anodal tDCS (a-tDCS) on the locus coeruleus (LC) via the trigeminal nerve, which is responsible for transmitting sensory information from the face, including touch, pain, temperature, and proprioception (Tramonti Fantozzi et al., 2021). The LC is located in the dorsal–rostral pons and contains neurons that densely innervate the thalamus and amygdala and sparsely innervate the neocortex, hippocampus, cerebellum, and spinal cord with unmyelinated projections (Levitt and Moore, 1978; James et al., 2021). When organically or electrically stimulated, one of the resulting actions is the co-release of norepinephrine (NE) and dopamine (DA) from the LC terminals in the hippocampus, which enhances sustained attention (e.g., vigilance) by modulating neural excitability mood by regulating neurotransmitter balance, and memory consolidation by amplifying long-term potentiation to promote an essential component of working memory, spatial memory formation (Baddeley, 2012; Lemon and Manahan-Vaughan, 2012; Mather and Harley, 2016; James et al., 2021).

Future research should consider stimulating key cortical regions that send significant signals to the LC when activated by tDCS or rTMS, resulting in subcortical activation and modulation. These regions include the DLPFC, which influences executive function, attention, and working memory (Ulam et al., 2015; Sacco et al., 2016; Moussavi et al., 2019; Neville et al., 2019; Siddiqi et al., 2019; Rodrigues et al., 2020; Eilam-Stock et al., 2021; Franke et al., 2022; Quinn et al., 2022; Ulrichsen et al., 2022); the prefrontal cortex (PFC), including the dorsal medial PFC (dmPFC) and orbitofrontal cortex (OFC), which are crucial for attentional control and impulsivity; the ACC which plays a part in error recognition, arousal, and stress response (Hara et al., 2017); and the trigeminal nerve pathway, which when stimulated, has been shown in animal models to impact attention, mood, and memory through hippocampal pathways (Majdi et al., 2023a,b; Chen et al., 2024). Thus, a combined understanding of hormesis, cortical target sites of NIBS, and brainstem behavior (e.g., RAS and LC) explain the improvement of attention, mood, and memory post-ABI (Giordano et al., 2017).

### NIBS as a viable and effective approach for enhancing positive alterations in neuroplasticity through cortical excitability, rCBF, and white matter integrity

Neuroplasticity can be neuronal and non-neuronal and synaptic or non-synaptic and is impacted by the activity being used during stimulation (Middleton et al., 2014), unique pathophysiology of ABI (Rodrigues et al., 2020), cortical excitability (Ulam et al., 2015), total brain volume (Neville et al., 2019), rCBF (Hara et al., 2017), and structural integrity of WM (Kurtin et al., 2021; Zhou et al., 2021). Studies incorporating diagnostic imaging provide evidence that NIBS is an effective approach for enhancing positive alterations in neuroplasticity.

In particular, the degree of cortical activity and related outcomes is significantly impacted by NIBS’ target site; i.e., structures directly under stimulation were most sensitive to neuromodulation, and the montage of electrode placement impacted the overall effect (Ulam et al., 2015; Hara et al., 2017; McCambridge et al., 2018; Siddiqi et al., 2019; Franke et al., 2022; Quinn et al., 2022). With effects similar to synaptic long-term potentiation (LTP) (i.e., a natural brain mechanism that uses repeated signaling to strengthen communication between neurons, making it central to overall cognitive function), rTMS may enhance brain state in a partially predictable manner after ABI by either increasing or reducing connectivity dependent on target site(s) and modulatory intention (Siddiqi et al., 2019; Franke et al., 2022). For example, after reducing the connectivity between the rDLPFC and sgACC, there was greater connectivity in the default mode network (DMN) and dorsal attention network (DAN), indicating frequency is a primary consideration based on the desired outcome (Siddiqi et al., 2019).

Although there was little evidence that rTMS impacted WM connectivity to improve neuroplasticity (Rao et al., 2019), rTMS was found to impact the interhemispheric imbalance of rcBF (Hara et al., 2017). Specifically, HF-rTMS reduced perfusion (i.e., the passage of fluids) directly under the stimulation site. LF-rTMS had a cross-hemispheric impact on a large area within the affected brain hemisphere by increasing perfusion around the rTMS target contralesionally (Hara et al., 2017). These findings may indicate that partnering HF and LF-rTMS may promote a balanced response to the cascade of internal injury post-ABI. While HF managed intracranial pressure by reducing inflammation over the ipsilesional region, LF simultaneously increased circulation contralesionally, which helped redistribute fluids, improve oxygenation to healthy tissues, and support overall neuroplasticity.

Due to the alterations and reorganization of neural communication post-ABI, an individual may find cognitive functions taking more effort (Sacco et al., 2016; Quinn et al., 2022). The brain may reside in a state of chronic overactivity, resulting in significant increases in activity (hyperactivation) when required to complete a task, resulting in decreased performance due to increased energy expense. Evidence suggests coupling tDCS with neurorehabilitation therapies is a potential solution to address this hyperactivation (Ulam et al., 2015; Sacco et al., 2016; Quinn et al., 2022).

Depending on neurorehabilitation coupling, stimulation protocol, and montage, tDCS can impact the strength of connections between brain regions and increase or decrease perfusion immediately following treatment and after a follow-up period, potentially impacting neural activity in the targeted areas (Quinn et al., 2022). Furthermore, both anodal and cathodal stimulation amplify the underlying patterns of brain network activity set by the current cognitive brain state (Kurtin et al., 2021). Similar to rTMS, cortical excitability from tDCS may not be restricted to the location of the anodal electrode but extends to the cathodal site, indicating an interhemispheric effect (Ulam et al., 2015). This far-reaching capability of NIBS may create more opportunities for holistic interventions targeting both hemispheres simultaneously post-ABI. When a task was coupled with tDCS, FA increased, and reduced connectivity was observed in the targeted structures, demonstrating cortical reorganization and improved WM connectivity, which may amplify cognitive efficiency during task performance (Kurtin et al., 2021).

Finally, a-tDCS can alter theta, delta, and alpha waves, causing cumulative cortical excitability and resulting in the possibility of both immediate and positive changes over time (Ulam et al., 2015).

### Behavioral outcomes using NIBS as a standalone or coupled intervention

Using a Coupled tDCS is more prevalent than rTMS, though both methods are efficacious in improving neuropsychological (Ulam et al., 2015; Sacco et al., 2016; Hara et al., 2017; Rodrigues et al., 2020; Eilam-Stock et al., 2021; Rushby et al., 2021; Franke et al., 2022; Quinn et al., 2022), neuropsychiatric (Sacco et al., 2016; Hara et al., 2017; Rao et al., 2019; Siddiqi et al., 2019; Rodrigues et al., 2020; Eilam-Stock et al., 2021; Rushby et al., 2021; Quinn et al., 2022; Ulrichsen et al., 2022), somatic (Eilam-Stock et al., 2021; Quinn et al., 2022; Ulrichsen et al., 2022), and motor outcomes (Middleton et al., 2014; Hara et al., 2016; McCambridge et al., 2018). In all cases, improved motor function resulted from coupled NIBS (Middleton et al., 2014; Hara et al., 2016; McCambridge et al., 2018), and outcomes were impacted by lateralization, contralesional or ipsilesional target site, and coupled intervention. All tDCS and rTMS studies coupled with cognitive therapy used computer-assisted programs (CAPs) for cognitive training (7 studies) (Sacco et al., 2016; Hara et al., 2017; Eilam-Stock et al., 2021; Kurtin et al., 2021; Rushby et al., 2021; Quinn et al., 2022; Ulrichsen et al., 2022). Using CAPs leads to greater motivation, better performance, and overall greater effect than offline paradigms (de Luca et al., 2014; Hill et al., 2016). Although there is an effect of CAPs as a standalone intervention (Kaldoja et al., 2015; Han et al., 2018), the efficacy of a-tDCS with CAPs on working memory (Sacco et al., 2016; Eilam-Stock et al., 2021; Quinn et al., 2022), EF (Sacco et al., 2016; Eilam-Stock et al., 2021), somatic function (e.g., fatigue, somatization) (Eilam-Stock et al., 2021; Quinn et al., 2022; Ulrichsen et al., 2022), and mental health (Sacco et al., 2016; Hara et al., 2017; Eilam-Stock et al., 2021; Quinn et al., 2022; Ulrichsen et al., 2022) supports a coupled intervention approach. rTMS is more widely used as an efficacious standalone approach for ABI (Leung et al., 2016; Hara et al., 2017; Neville et al., 2019; Rao et al., 2019; Siddiqi et al., 2019; Rodrigues et al., 2020; Franke et al., 2022). Like coupled tDCS, standalone rTMS has improved many symptoms post-treatment, resulting in a near-effect on neuropsychological, neuropsychiatric, and physical symptoms (Asloun et al., 2008; Bikson et al., 2010; Eldaief et al., 2013; Chang et al., 2023; Evancho et al., 2023; Zotey et al., 2023; Calderone et al., 2024). Similarly, studies measuring the longevity of intervention noted that EF, mental health, and somatic symptoms were maintained or improved by the follow-up period (between 1 week and 1 month) (Leung et al., 2016; Rodrigues et al., 2020; Franke et al., 2022). This improvement may be due to the far effect of rTMS on delta power, which is strongly correlated to EF and depression (Franke et al., 2022). These findings support using standalone rTMS to improve symptoms with both a near and far effect. Two studies investigating rTMS on DAI denied significant effects on neuropsychological and neuropsychiatric outcomes, regardless of standalone or coupled status (Hara et al., 2017; Neville et al., 2019). Although this could have been due to frequency and target site, it is worth considering that progressive atrophy resulting from primary and secondary axotomy and microhemorrhages associated with DAI impacts the effectiveness of rTMS as opposed to other ABI (Neville et al., 2019).

### NIBS potential as the primary mechanism of change and future directions

Though NIBS has been investigated for decades, the change mechanism is not yet fully understood. To support comprehensive research on neuromodulation’s influence on behavior, alternative explanations for change should be determined to improve understanding and establish future directions. This section explores the capacity of NIBS as the direct mechanism of change while considering that the mechanisms of action underlying stimulation may not be sufficient for explaining behavior outcomes and there may be other coexisting variables influencing behavioral change post-stimulation, including (1) depression, (2) attention, (3) placebo effect, and (4) widespread activation within cortical modules beyond the target site.

### Improvement of depression

Depression and anxiety impact working memory, attention, speed of information processing, and executive function (Priyamvada et al., 2015; Uiterwijk et al., 2022), thus generating the possibility that improving mood symptoms may result in improved behavioral outcomes. However, many tDCS and rTMS studies have found positive isolated effects of NIBS on short—and long-term behavioral outcomes after accounting for depression and anxiety (Ulam et al., 2015; Leung et al., 2016; Rodrigues et al., 2020; Franke et al., 2022). One study investigated a-tDCS over the left DLPFC and controlled for symptoms of depression and anxiety and concluded that statistically significant favorable results on clinical outcomes were due to the modification of electrophysiological frequencies (i.e., increased alpha waves and decreased delta and theta waves) (Ulam et al., 2015). Another coupled tDCS study used two anodal leads on bilateral DLPFC with cognitive training to investigate the effects of tDCS on divided attention. This study excluded patients with neuropsychiatric illness The study found statistically significant results for divided attention while the change on the depression scale was insignificant (*p* = 0.305) (Sacco et al., 2016).

Currently, the data are limited to draw substantial conclusions regarding NIBS’s ability as the primary mechanism of change; however, emerging studies have shown that NIBS has the potential to engage the primary target if the stimulation protocol is being followed properly e.g., correct cortical positioning of the electrode using brain mapping systems (Antal et al., 2017) and the confounding variables such as depression/anxiety are accounted for either at the study design or the analysis level. Future research should take measures to control for the presence of mood symptoms and incorporate neuroimaging assessments to enhance confidence in NIBS’s ability to act as the primary mechanism of change.

### Improvement of attention

Attention is often the first function to be addressed in cognitive therapy following ABI due to its influence on other neuropsychological functions (i.e., concentration, short-term memory, vigilance, and working memory) and its mediating role between information processing and language (Lee et al., 2023). Evidence suggests these functions can be improved by cognitive therapy, NIBS, or both (Leung et al., 2016; Hara et al., 2017; Quinn et al., 2022; Lee et al., 2023). Still, the mechanism of change to support these outcomes continues to be explored through improved understanding of target specificity and cortical excitability.

Many rTMS and tDCS studies investigate the effectiveness of neuromodulation to improve attention, working memory, and other neuropsychological functions that may be impacted following an ABI by attempting to specifically target the dorsal regions of the ACC and DLPFC for their role in selective attention, working memory, performance reappraisal, and monitoring (Clarke et al., 1969; Ochsner et al., 2012; Leung et al., 2016; Hara et al., 2017; Kurtin et al., 2021; Quinn et al., 2022). However, the barrier to target specificity is confounded by the complexities of attention and its relationship to other neuropsychological functions, making it difficult to know which function most influences overall outcomes (Lee et al., 2023).

Kurtin et al. (2021) suggest the improvement of cognitive efficiency and behavioral outcomes is a byproduct of NIBS’ ability to improve cortical organization and WM connectivity (Kurtin et al., 2021). This is substantiated by Hebbian theory, which suggests that sustained application of stimulus for more than several minutes leads to increased synaptic activity, thereby improving the receptiveness of the second area to respond to input from the first area (i.e., improvement of LTP) (Siddiqi et al., 2019; Korai et al., 2021; Pitcher et al., 2021; Barbati et al., 2022; Franke et al., 2022). Although intricacies are involved in directly mapping the role of NIBS on behavioral outcomes in studies involving attention, determining neural correlates of attention modulation may provide insight into NIBS capacity as a primary or mediatory agent of change(Nani et al., 2019). Additionally, studies seeking to elucidate the role of attention in improved performance after NIBS should consider specific contributions and mechanisms of action of attention to other neuropsychological functions to better understand its influence on outcomes.

### Placebo effect

A placebo effect is a psychobiological phenomenon occurring in the brain after administering an inert substance or sham physical treatment (Price et al., 2008; Benedetti et al., 2011). Some studies have investigated this effect as the primary reason for positive outcomes after NIBS to account for inconsistencies among active and sham stimulation groups (Conforto et al., 2014; Braga et al., 2021). For example, one study using tDCS over the left DLPFC used neuropsychological tests to assess working memory, attention, and general executive function and discovered that both active and sham groups experienced significant improvements (Ulam et al., 2015). A possible explanation for this phenomenon is expectation and reward motivation (Ballard et al., 2011).

The expectancy theory suggests that non-volitional responses such as pain perception, emotional reactions, and other sensations can be self-confirming (Kirsch, 1985); that is, the mere suggestion that NIBS is effective can elicit or enhance the occurrence of those desired results (Kirsch, 1985). Expectations (i.e., the belief in the likelihood of something happening) can modulate a variety of cognitive processes, including perception (de Lange et al., 2018), motor control (Weinberg et al., 1979), and working memory (Bollinger et al., 2010). One coupled study combining tDCS and motor training investigated the likelihood of a placebo effect in tDCS and whether the participants’ beliefs about tDCS impacted this effect (Haikalis et al., 2023). The active and sham tDCS groups showed more improvement on the motor task than the control group, indicating a significant placebo effect of tDCS on motor learning (*p* = 0.007); furthermore, those with higher beliefs and expectations of tDCS improved more than those with lower beliefs and lower expectations (Haikalis et al., 2023).

Expectation also has a psychobiological effect on cortical activation and dopaminergic distribution, which may impact neuropsychological and physical symptoms following ABI (Conforto et al., 2014; Braga et al., 2021). In one study, both active and control groups demonstrated improvements in executive function and post-traumatic symptoms from baseline to 1 month, which significantly correlated with the degree of connectivity change between the right DLPFC, which influences the physiology supporting motivated behavior, attention, and memory(Ballard et al., 2011), and the left anterior insula which has a primary role in the representation of natural rewards and their integration with attention and emotions (*p* = 0.02) (Haaranen et al., 2020). As seen in post-traumatic migraines, the placebo effect does not only impact neuropsychological functions but may also influence physical improvements. Ebell et al. (2004) found that sham rTMS over the DLPFC for 8 weeks induced a greater amelioration of post-concussive headaches compared to active rTMS, with a decrease in the number of headache days greater than 50% in the sham group, suggesting a powerful placebo response. Just as the impact or reward motivation on the mesocortical dopaminergic regions (Ballard et al., 2011), this sham treatment may have potentiated placebo analgesia by increasing patients’ expectations, thereby inducing dopamine release (Conforto et al., 2014; Braga et al., 2021).

The placebo effect produces physiobiological results, which may indicate it can be exploited for clinical benefit while also accounting for improvements among active and sham groups (Annoni, 2013). Future studies may support this role by analyzing expectation priming (Rabipour et al., 2018), silencing the expectation mechanism by optimizing the placebo parameters (Benedetti et al., 2011), and using specific assessments to quantify participants’ prior beliefs and expectancies related to NIBS at varying points throughout the assessment, intervention, and follow-up periods (Braga et al., 2021).

### Target specificity

Finally, a challenge in establishing the clear effectiveness of neuromodulation to improve behavioral outcomes arises from the variability in how it impacts both proximal and distal brain areas relative to the target site. Neuromodulation may indirectly modulate other unnecessary or antagonistic cortical modules, thereby confounding NIBS’s true mechanism of action to influence behavioral change (Morya et al., 2019). Improvements in technology and electrophysiological understanding may offer a solution to improving target specificity.

Due to its ability to indirectly measure the brain’s neural activity and allow for millimeter precision in establishing target sites through its high spatiotemporal resolution (Kim et al., 2021), structural MRIs are used to design participant-specific tDCS or TMS protocols (Rezai et al., 2016; Sacco et al., 2016; Chudy et al., 2018; McCambridge et al., 2018; Rao et al., 2019; Siddiqi et al., 2019; Kurtin et al., 2021; Raguž et al., 2021; Quinn et al., 2022). Although initially using fMRI is shown to improve stimulation site specificity, functional connectivity and EEG may be used to establish a more optimized intervention design, including electrode placement, frequency, dose, and duration (Boes et al., 2018).

The phenomenon of ephaptic coupling and the use of the Ephaptic Modulation Index (EMOD) can explain the mechanisms of action underlying low-focality and broad neuromodulatory impact of NIBS while also offering a potential solution for optimizing future preliminary assessment, stimulation protocols, and positioning (Morya et al., 2019; Ruffini et al., 2020). Ephaptic coupling postulates that when populations of neurons synchronize their activity, it produces substantial electric fields (Ruffini et al., 2020). These fields can influence the excitability of appropriately oriented neurons, regardless of distance (Jefferys, 1995). Neuronal circuits are sensitive to weak endogenous or exogenous low-frequency (0–100 Hz) electric fields (> 0.1 V/m) (Ruffini et al., 2020). tDCS delivers these weak current waveforms and thus is expected to produce spatially extended electric fields throughout the brain (Ruffini et al., 2013, 2020). For example, Ulam et al. (2015) used tDCS, resulting in brain activations that extended from the anodal to cathodal electrodes across hemispheres (Ulam et al., 2015). The same is true for rTMS, as seen in a study that demonstrated the long-term impact of inhibitory LF-rTMS targeting the contralesional motor cortex post-stroke. The results indicated increased cortical excitability on the ipsilesional site, leading to statistically significant improvement in upper limb performance on the box and block test (*p* = 0.049) and MEP amplitude of both paretic (*p* = 0.002) and non-paretic (*p* = 0.035) hands (Luk et al., 2022).

EMOD can support future studies as it accounts for endogenous electric field strength, neuronal excitability, brain topography, spatial relationships, and frequency of neuronal activity within each cortical module (Ruffini et al., 2020). Behavioral modification by tDCS and rTMS depends on the relationship between neuronal signals required for a particular task and the noise generated by the stimulus (i.e., neuronal activity non-specific for the cortical module being targeted or the activity being conducted) (Miniussi et al., 2013; Braga et al., 2021). By accumulating knowledge of the natural state of electrical fields within each cortical module during the assessment period, future researchers in this area may better understand neuronal qualities and neuromodulation response potential (Ye et al., 2022), design protocols based on informed knowledge of potential summation of endogenous and exogenous fields, and which modules may have reduced impact potential based on ephaptic communication (Ruffini et al., 2020). More research is needed in ephaptic coupling and EMOD as it relates to NIBS in ABI to demonstrate its capacity to improve target site selection and stimulation application.

### Strengths and limitations

This review has several strengths, including the representation of current research exploring the efficacy of NIBS in both standalone and coupled designs, the discussion on the potential of NIBS as the primary mechanism of change, and the exploration of a potential mechanistic model of neuroplasticity to explain the impact of NIBS on attention, memory, and mood. Additionally, it examines alternative explanations for the positive outcomes associated with NIBS and provides future recommendations that may benefit clinicians and researchers using NIBS in clinical trials. To the best of our knowledge, this is the only review that has addressed multiple aspects of NIBS including mechanism and efficacy within a single article. Moreover, while the review focuses on ABI, the explanation of the NIBS mechanism could also be helpful for other neurological disorders.

The limitations of this review could be addressed in future studies, including a more detailed exploration of the locus coeruleus concerning TBI and NIBS as well as further research based on cognitive science methodologies to understand common post-ABI symptoms such as information processing, attention, and working memory, and how these relate to the mechanisms of action presented.

## Conclusion

rTMS and tDCS may improve neuroplasticity and behavioral outcomes after ABI. Although rTMS is used more widely as an efficacious standalone intervention, tDCS has emerged as another non-invasive approach to couple with neurorehabilitation therapies. The mechanistic model of neuroplasticity for NIBS suggests that these techniques promote synaptic plasticity and modulate neural network activity through controlled, low-level stimulation, which can induce beneficial neurochemical and structural changes in the brain. More research is needed to better understand the role of NIBS as the primary mechanism of change and its potential to positively alter symptoms after ABI.
